# Structural Insight into Non-Enveloped Virus Binding to Glycosaminoglycan Receptors: A Review

**DOI:** 10.3390/v13050800

**Published:** 2021-04-29

**Authors:** Marie N. Sorin, Jasmin Kuhn, Aleksandra C. Stasiak, Thilo Stehle

**Affiliations:** 1 Interfaculty Institute of Biochemistry, University of Tuebingen, 72076 Tuebingen, Germany; marie.sorin@univ-nantes.fr (M.N.S.); jasmin.kuhn@uni-tuebingen.de (J.K.); aleksandra.stasiak@uni-tuebingen.de (A.C.S.); 2 Faculté de Médecine, Université de Nantes, Inserm, Centre de Recherche en Transplantation et Immunologie, UMR 1064, ITUN, F-44000 Nantes, France; 3 Department of Pediatrics, Vanderbilt University School of Medicine, Nashville, TN 37232, USA

**Keywords:** viruses, glycans, glycosaminoglycans, glycovirology, non-enveloped viruses, structural biology

## Abstract

Viruses are infectious agents that hijack the host cell machinery in order to replicate and generate progeny. Viral infection is initiated by attachment to host cell receptors, and typical viral receptors are cell-surface-borne molecules such as proteins or glycan structures. Sialylated glycans (glycans bearing sialic acids) and glycosaminoglycans (GAGs) represent major classes of carbohydrate receptors and have been implicated in facilitating viral entry for many viruses. As interactions between viruses and sialic acids have been extensively reviewed in the past, this review provides an overview of the current state of structural knowledge about interactions between non-enveloped human viruses and GAGs. We focus here on adeno-associated viruses, human papilloma viruses (HPVs), and polyomaviruses, as at least some structural information about the interactions of these viruses with GAGs is available. We also discuss the multivalent potential for GAG binding, highlighting the importance of charged interactions and positively charged amino acids at the binding sites, and point out challenges that remain in the field.

## 1. Introduction

Viruses are infectious entities that require a living organism, a host, to support their replication. They can target any kind of organism, ranging from complex hosts such as animals or plants to microorganisms such as bacteria or archaea. Until now, more than 6500 types of viruses have been discovered, and more than 260 of these can infect humans [1]. Viruses are encoded by DNA or RNA genomes, which are packaged into and protected by a protein capsid. Enveloped viruses additionally contain a lipid bilayer shell that is derived from the host cell membrane, while non-enveloped viruses lack such a membrane envelope and are encased in a capsid protein shell.

In order to infect a host organism and replicate, specific virus proteins interact with receptor molecules on the host cell surface. Among these receptors, carbohydrate-based structures, or glycans, are used by many viruses to infect cells [2]. Such glycans can be grouped into three major groups: structures that terminate in sialic acids (sialylated glycans), neutral structures that are based on histo-blood group antigens (HBGAs), and structures known as glycosaminoglycans (GAGs). These GAGs are long, linear, negatively charged polysaccharides with molecular weights ranging from 10 to 100 kilodaltons (kDa), and they can be divided into two main types: sulfated GAGs, including heparan sulfate (HS), heparin, chondroitin sulfate (CH), and dermatan sulfate (DS); and non-sulfated GAGs, including hyaluronic acid [3].

HS and heparin are both linear polysaccharides composed of repeating units of uronic acid linked to D-glucosamine. HS is ubiquitously found on the cell surface or on mammalian extracellular matrix proteins, where it is involved in multiple biological processes, while heparin is secreted by mast cells and is medically used as an anticoagulant. HS and heparin share the same backbone, which is comprised of uronic acid and D-glucosamine, where the uronic acid moiety can either be α-L-iduronic acid (IdoA) or β-D-glucuronic acid (GlcA). Both components of the polysaccharide can be modified by sulfatation, deacetylation, and epimerization during biosynthesis [4]. There are three main differences between heparin and HS: (i) The uronic acid in heparin is predominantly IdoA, while it is GlcA in HS. (ii) D-glucosamine is mainly N-sulfated in heparin, while it is N-acetylated in HS. (iii) Heparin is composed of at least 70–80% of Ido(2S)-(1→ 4)-GlcNS(6S) disaccharides, while in HS 40–60% of the disaccharide units are GlcA-(1→ 4)-D-glucosamine, either N-acetylated or N-sulfated. Taken together, these differences indicate that heparin carries more sulfatation, and therefore has a higher overall negative charge compared to HS [5]. Chondroitin sulfate is also a linear polysaccharide composed of repeated units of uronic acid and N-acetylgalactosamine (GalNAc). Similar to HS, CS is found on the cell surface and in the extracellular matrix, and is involved in diverse physiological processes [6]. Dermatan sulfate, also known as chondroitin sulfate B, is the main GAG expressed in skin tissue. DS is structurally close to CH because it is composed of GalNAc, but also to HS and heparin because of the presence of IdoA in the disaccharide repeating unit [7].

While several non-enveloped viruses have been found to use GAGs as their receptors (e.g., adenoviruses [8] and rhinoviruses [9]), these results have not necessarily been verified in vivo, and it remains unclear whether the reported receptor specificity is the result of cell culture adaptation or is also present in nature [10]. This review aims to provide an update on the structural work characterizing the interaction of non-enveloped viruses with sulfated glycosaminoglycans, especially heparin. Among the structural studies that will be discussed, only structures with a resolution better than 4 Å will be considered in detail, and primarily only ones where electron density maps for the ligands are publicly available and can be inspected to assess the quality of the ligand fitting.

## 2. Adeno-Associated Virus

**Figure 1:**
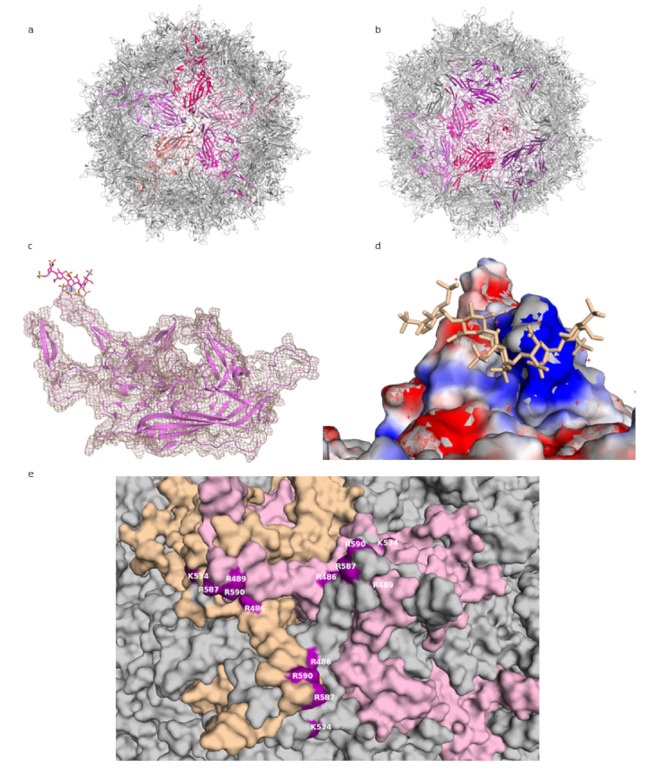
AAV-DJ capsid and its binding by GAG (PDB: 5UF6). (**a**) The viral capsid, with one constituting pentamer highlighted in shades of purple. (**b**) The viral capsid, with one constituting hexamer, and a threefold symmetry axis peak, highlighted in shades of purple. (**c**) A close-up of the density (salmon) for the structure at σ 1.5, showing that the capsid protein (purple) matches it closely and can be well resolved, while the ligand (raspberry) density is not visible at this level. (**d**) The charge distribution in the proximity of the threefold peak. Negatively charged residues are shown in red, positively charged in blue, ligand in wheat. (**e**) Close-up of the residues identified in mutational studies as contributing to binding HS, highlighted in purple. Capsomers in wheat, pink, and grey. Figures were created using PyMOL [23].

Adeno-associated viruses (AAVs) are single-stranded DNA viruses, members of the family *Parvoviridae*, with a small genome of about 4.5 kilobases. They require the host cell to be infected by another virus, typically an adenovirus, for productive replication, and they are not associated with human pathology. Their significance in research is connected to their potential as gene delivery vectors, associated with, among others, their ability to transduce non-dividing cells and lack of pathogenicity. The AAV capsid is about 26 nm in diameter, composed of 60 subunits, and has T = 1 icosahedral symmetry (see Figure 1a) [11]. The three capsid proteins, VP1, VP2, and VP3, all share most of the C-terminal sequence. Compared with the other two proteins, about ten times more copies of VP3 are found in the capsid. All three proteins consist of a jelly roll β-barrel fold, with loops between the barrel strands containing most of the sequences that differ among AAV strains and that are mainly responsible for receptor binding [11]. The loops form the triple-capsomer peaks close to the threefold symmetry axis (Figure 1b)—a location where multiple residues associated with heparin binding cluster [12,13,14].

While heparan sulfate was long considered to be the primary receptor for AAVs, recent research suggests that this role is in fact performed by the adeno-associated virus receptor [15], with GAGs serving as lower-affinity co-receptors of significance for attachment. Structural studies of GAG binding to AAV generally have suffered from difficulties in obtaining well-diffracting crystals of the virus–receptor complex, either by co-crystallization or soaking [16]. These difficulties have excluded X-ray crystallography as a method for the structural analysis of AAV–GAG complexes, and led to efforts of obtaining cryoelectron microscopy (cryoEM) structures instead.

Among the first structures of AAV–GAG complexes are two examples that were solved before the cryoEM “resolution revolution” [17], at lower resolutions of 18 Å [18] and 8 Å [19]. Both reports examined the structures of AAV type 2 in complex with heparin, and came to opposite conclusions, with the lower-resolution analysis postulating significant conformational shifts on GAG binding that were not observed in the higher-resolution structure. Additional studies of complexes of AAV-DJ (a mutant strain selected for resistance to most common human neutralizing antibodies, with a high capsid sequence similarity to AAV-2) at 5 Å [16] and 2.8 Å resolution [20] provided data that argue against a large conformational shift. In these cases, heparin analogues were used to mitigate the issues caused by multiple-site, asymmetric binding of the physiological ligand and to provide a clearer view of binding.

While the use of smaller, shorter GAG analogues such as fondaparinux (five sugar moieties) or sucrose octasulfate (SOS) is a promising approach to the problem of averaging out the non-icosahedral ligand density, the resulting structures do not provide high-resolution information on GAG binding to AAV. In the lower-resolution structure at 5 Å, the level of molecular details is low (as expected at this resolution), and while electron density for the ligand is visible, this density is largely globular and does not allow the assignment of specific contacts. Unfortunately, this situation is not improved for the 2.8 Å structure, probably due to low occupancy of the ligand. The viral capsid density allows for a clear delineation of the main chain and most side chains of the amino acids, but the ligand electron density is not detailed enough to reliably place the ligand or to deduce much information about its conformation (see Figure 1c).

The most detailed structural information on GAG binding to AAV is provided not through structural analyses but mutational studies. The residues identified by these studies as necessary (or of importance) for heparin binding can then be mapped onto the capsid or capsomer structures, where they do localize in the proximity of ligand densities from structural studies. Thus, positively charged residues at the threefold peak, primarily arginines (R587 and R590 from one capsomer and R486 and R489 from another—see Figure 1e; residue numbering according to AAV-DJ sequence), have been shown to play an important role in the cell binding of AAV-2, since a loss in infectivity was seen after their mutation [12]. These residues, along with others, form a positively-charged patch coming downwards from the peak (Figure 1d). GAG binding is often mediated by positive charges on the protein, with positively charged residues forming ionic interactions with the sulfur groups, and this is where the GAG density is found in the structures. Structural and mutational studies of AAV-6, a strain which binds both HS and sialic acid, have also highlighted the threefold peak as the location of an HS binding site [13,14]. Given that GAG molecules can achieve significant lengths, it is possible that one chain would engage multiple sites on a single capsid, winding in the clefts between the peaks. This would serve to increase the binding affinity and enhance the strength of the interaction, increasing the chance of entry.

Structural studies of AAV binding to GAGs form an interesting example of the challenges encountered in this field, and of characterizing interactions despite the unavailability of a high-resolution structure of the complex. Combining the high-resolution apostructures of AAV with extensive mutational data, an overview of the binding site locations and their residues has been obtained. A recent cryo-EM structure of AAV-DJ at a resolution below 1.6 Å [21], and a continuing interest in AAV as a gene therapy vector [22], shows this is an active field, both in structural biology and applied medicine; therefore, elucidating the details of AAV–GAG interactions at atomic (or near atomic) resolution would be an important development.

## 3. Human Papillomavirus

Human papillomaviruses (HPVs) are small, non-enveloped viruses containing a double-stranded DNA genome. Over 170 HPV types have been described, of which approximately 40 can be transmitted by sexual contacts, making HPV the most common sexually transmitted infection (STI) [24]. It is estimated that more than 80% of the world’s population has at one point been in contact with HPV, although 90% of infections spontaneously resolve within the first two years and are often asymptomatic. However, some strains can cause warts or precancerous lesions (progressing to cancer), mainly of the genital or oropharyngeal tract. Strains HPV6 and HPV11 are the most common cause of genital warts, while 70% of cervical cancers are associated with HPV16 or HPV18 [25,26].

**Figure 2:**
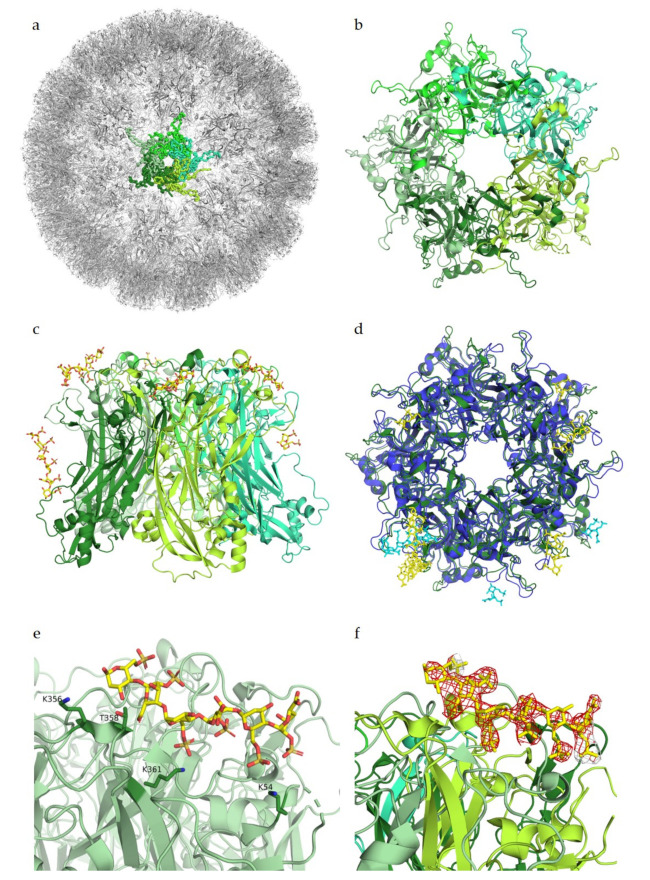
Glycan engagement of HPV16. (**a**) Capsid structure of HPV16 L1 pentamers (PDB ID 5KEP). L1 subunits of one pentamer are displayed in different shades of green. The highlighted pentamer is represented as a ribbon, the remaining capsid is depicted as cartoons. (**b**) Top view of a HPV16 L1 pentamer (PDB ID 5W1O). Subunits are displayed in cartoon representation and colored in different shades of green. (**c**) Side view of the pentamer. The heparin ligand is colored by atom type and represented with sticks in yellow. (**d**) Superposition of HPV16 L1 pentamer in green with its heparin ligand in yellow with HPV18 L1 pentamer (PDB ID 5W1X) in blue and its ligand in cyan. (**e**) Close-up view of one ligand binding site of HPV16. Ligand-binding residues K356, T358, K361, and K54 are represented as sticks. (**f**) Close-up view as in (**e**) of one ligand binding site with the negative F_o_–F_c_ difference map of the ligand contoured at 3σ, colored in red. Figures were created using PyMOL [23].

The HPV capsid is comprised of 360 copies of the major capsid protein L1 arranged in 72 capsomers of L1 pentamers, forming an icosahedral T = 7d geometry (Figure 2a,b). The minor capsid protein L2 is incorporated into the viral capsid in a not fully understood manner. The cell entry of HPV is mediated by binding of the viral capsid to cell surface proteoglycans as the primary receptors [27,28,29].

Heparin binding assays identified largely positively charged peptide sequences at the C-terminus of L1, spanning amino acids G472 to L505, as the main interaction partners for GAGs [30]. Additional interaction studies determined similar residues of L1 as well as residues of L2 to bind to heparin, with binding constants in the high millimolar range [31]. However, both studies were only performed with small peptide fragments of both L1 and L2. Studies by Dasgupta et al. (2011) for the first time revealed structural details of the interactions between HPV and receptor candidates. Using co-crystallization experiments with heparin, these studies showed multiple heparan sulfate binding sites in the intact pentamers of both HPV16 and HPV18, demonstrating that these two strains display somewhat different oligosaccharide binding patterns (Figure 2d). The study identified key regions for ligand binding on surface loops (HI, FG, BC, EF, and a4) of the capsid protein L1, which are conserved between HPV16 and HPV18. These regions were determined to be located on the capsid-distal sides as well as on the capsid-proximal sides of the pentamer (Figure 2c). As more data are available for HPV16, we will mainly focus on HPV16 from here on. The crucial residues interacting with the negatively charged heparin in the HPV16 binding sites were identified as positively charged lysines that engage in charge–charge interactions with the sulfated groups of the receptors. These lysines form a mostly positively charged binding pocket in comparison to the highly negatively charged pore. For the two binding sites on the capsid-distal side of the pentamer, interaction partners were determined to be K54, K356, and K361 (Figure 2e), as well as K278 and N285 for a second binding site. Additionally, polar residues T358 and T266 were found to contact the glycan through polar interactions. To our knowledge, the characterized residues had not been identified before in binding assays as involved for glycan binding. Mutational experiments by Dasgupta et al. have confirmed K278 and K361 as essential residues for HPV16 pseudoviral infection, while mutations of the other structurally identified binding partners did not significantly influence infectivity [32].

The structure of HPV16 in complex with heparin has a resolution of 2.8 Å, meaning that residues and ligands should be clearly visible in the electron density, as previously described for adeno-associated viruses. In the difference map (F_o_–F_c_ map, from PDB ID 5W1O), no negative difference density can be observed for the L1 pentamers; however, for the complex structure, a large amount of negative electron density around the heparin ligand is visible (Figure 2f). This negative density indicates that the modelled ligand structure does not fully agree with the experimentally observed data, perhaps because of high flexibility of the glycan or low occupancy, as both would show density in the initial map but would display negative density after refinement. Nevertheless, it is also possible that the glycan was placed incorrectly in the structure. Structural studies by Guan et al. (2017) identified a different heparan sulfate binding site, which overlaps with two of the previously identified binding sites [33]. However, no complex structure or difference maps are available for the newer study, and therefore cannot be considered for this review.

As already discussed for AAV complex structures, complex structures of HPV with heparin or HS published to date should be treated with caution, as the available electron density maps do not allow for unambiguous assignment of contacts between protein and ligand. It also becomes clear that flexibility and occupancy represent major challenges in structural elucidations of virus–GAG interactions.

## 4. Polyomavirus

The *Polyomaviridae* form a family of non-enveloped icosahedral dsDNA viruses. To date, fourteen human polyomaviruses have been identified. Infection mostly occurs during childhood, and the viruses typically persist in host cells asymptomatically. In the context of immunosuppression, polyomavirus family members can reactivate and lead to various diseases [34]. The BK (BKPyV) and JC (JCPyV) polyomaviruses were the first polyomaviruses to be discovered, and they induce polyomavirus-associated nephropathy and progressive multifocal leukoencephalopathy in immunocompromised patients, respectively [35,36]. The more recently discovered Merkel cell polyomavirus (MCPyV) was found in Merkel cell carcinomas, and is the first confirmed human oncovirus within the *Polyomaviridae* family [37]. Similar to papillomaviruses, polyomaviruses have a capsid composed of 72 pentameric capsomers (referred to as VP1 pentamers) with an icosahedral T = 7d symmetry. However, polyomaviruses are smaller, with a diameter of around 50 nanometers compared to 60 nanometers for papillomaviruses. Moreover, the structures of the L1 and VP1 differ significantly. VP1 capsomers have a barrel-shaped morphology while papillomavirus L1 capsomers are mushroom-like protrusions with star-shaped pentamers [38].

**Figure 3:**
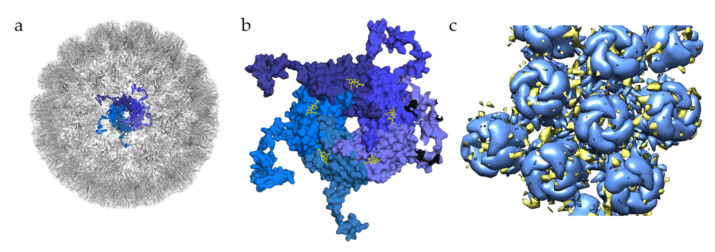
Glycan engagement of BKPyV. (**a**) Capsid structure of BKPyV (PDB ID 6ESB). The capsid is represented in the gray cartoon, except for the highlighted pentamer, where VP1 monomers are displayed as ribbons in different shades of blue. (**b**) Top view of BKPyV VP1 pentamers in interaction with GT1b. The pentamer is displayed as a surface colored in different shades of blue for each subunit. The double sialic acid part of GT1b is represented as yellow sticks. (**c**) Surface representation of BKPyV VP1 pentamers, in blue, associated with the difference map for heparin, in yellow (generated through subtraction of the unliganded VLP map (EMD-3946) from the heparin-VLP map (EMD-3945)). Figures were created using PyMOL [23] and Chimera [51].

When initiating infection, many polyomaviruses use glycans terminating in the sialic acid N-acetyl neuraminic acid (Neu5Ac) as their receptors. Interactions are mediated through surface loops at the outer margin of the major capsid protein VP1, and structural information is available for many complexes of polyomavirus VP1 pentamers with their cognate receptors [39,40,41,42,43,44,45]. These analyses have revealed that polyomaviruses differ in their specificities for sialylated glycan structures. For example, BKPyV binds to cells via sialylated structures found in b-series gangliosides, which carry two sialic acids on the inner galactose (Neu5Acα2-8Neu5Acα2-3Gal) (Figure 3b) [41]. On the other hand, JCPyV specifically engages glycans carrying terminal α2,6-linked Neu5Ac in the context of the monosialylated lactoseries tetrasaccharide c (LSTc) glycan [45]. MCPyV binds to yet another type of sialylated glycan carrying a terminal Neu5Acα2-3Gal motif [40].

For some polyomaviruses, additional interactions with non-sialylated glycans have been reported to be required for cell entry. For example, MCPyV requires both GAGs and sialylated glycans for entry, and these interactions occur sequentially, with GAGs serving as the primary receptor [46]. BKPyV and JCPyV have also been proposed to bind GAGs [47]. This recent study showed only a small decrease in the cell attachment of wild-type JCPyV and BKPyV virus-like particles (VLPs) on cells producing non-sialylated or hyposialylated glycans [47]. This result indicates that these two viruses do not require sialylated glycans for initial binding to cells. Further work showed that simultaneous removal of GAGs and sialylated glycans drastically reduced the binding of those VLPs to cells. Taken together, the results of this study suggest that wild-type BK and JC viruses can interact with either GAGs or sialylated glycans for initial cell attachment [47].

In the case of BKPyV, a structural study has investigated interactions of this virus with GAGs, solving the structure of BKPyV virus-like particles via cryo-electron microscopy (cryoEM) at 3.8 Å resolution. As expected, the BKPyV capsid was found to consist of 72 pentamers of the major capsid protein VP1 arranged in T = 7d icosahedral symmetry (Figure 3a). Six distinct VP1 conformations, all sharing the β-sandwich jelly roll fold, were found within the asymmetric unit of the capsid. The C-terminal arms are well resolved, and the structure of the assembled VLP shows how they are extended into adjacent capsomers and maintain a stable capsid structure, similar to what has been observed for other polyomavirus family members [39,48]. A structure of BKPyV VLPs in complex with heparin was also determined at a resolution of 3.6 Å. No structural differences at the protein level were seen between this structure and the unliganded BKPyV structure, establishing that heparin binding does not induce a structural change of the capsid. However, electron density corresponding to heparin was not visible at high resolution, and difference maps calculated to identify potential binding sites had to be computed at lower resolution, yielding a rather imprecise representation of the BKPyV–heparin complex at only 8 Å resolution. Difference density was observed between capsomers as well as at the top of each capsomer (Figure 3c). Density in between pentamers cannot be associated with GAG binding with certainty. However, this site is positively charged, which would fit the observations made for other viruses where GAG binding sites are indeed positively charged. The density observed in the pore of the pentamers could be attributed to the effects of symmetry averaging, but the authors do not completely discount the idea of GAG binding to the pore [49].

A detailed study conducted in 2011 investigated MCPyV cellular tropism and found that this virus sequentially engages GAGs and sialylated glycans. Through multiple binding and transduction assays, it was shown that the initial attachment of MCPyV to cells requires GAGs such as HS and CS. Particularly, N-sulfated and/or 6-O-sulfated forms of HS are needed for infectious entry of the virus [46].

Structural information on GAG interactions with MCPyV is currently limited to a study by Bayer et al., which analyzed the binding of different GAGs to MCPyV particles and isolated VP1 capsomers with saturation transfer difference nuclear magnetic resonance spectroscopy (STD NMR). Interestingly, it was found that isolated, unassembled VP1 pentamers did not bind any of the tested GAGs, while the assembled virus capsid gave a strong binding signal. Isolated VP1 pentamers are thus not able to establish interactions with GAGs, in contrast to the interactions with sialylated glycans, which were analyzed using VP1 pentamers. Additional STD NMR experiments showed that MCPyV VLPs were able to bind HS-derived disaccharide, heparin-derived octosaccharide, DS-derived hexasaccharide, as well as the HS-derived pentasaccharide Arixtra(fondaparinux). Based on these observations, it is clear that MCPyV can bind a broad range of GAGs, and that the presence of sulfatation seems to be essential for interaction. Most importantly, GAG binding by MCPyV requires more than one VP1 pentamer, suggesting that the GAG binding sites most likely lie in the recessions that separate individual VP1 pentamers. However, attempts to visualize this interaction by solving the structure of MCPyV VLPs with GAGs through crystal soaking or cryoEM studies were not conclusive [50].

## 5. Conclusions

In this review, we surveyed the available structural data on the binding of GAGs to non-enveloped viruses. It is clear that GAGs are exceedingly important glycans that play key roles in the entry processes of many viruses, including several that are relevant to human health. However, reliable and detailed structural information about contacts between GAGs and virus capsid proteins is essentially non-existent. More than anything, our review has therefore highlighted the challenges that hamper structural studies of GAG receptor binding to viruses. Crystallographic methods are in many cases unsuitable, primarily due to the difficulty of obtaining crystals—particularly with asymmetric, heterogeneous molecules such as HS. This moves the focus to cryo-EM. While it is developing rapidly, this method does not yet necessarily match crystallography’s resolution and suffers from a paucity of reliable metrics for assessing structure quality, particularly because of the variety of workflows applied for data processing, which can significantly influence the result. Perhaps more importantly, in the case of virus–GAG interactions, the icosahedral symmetry imposed during processing can result in the averaging out or weakening of the signal of the non-icosahedrally symmetrical ligand. Issues with ligand heterogeneity, and ligand binding to multiple sites on the same capsid also increase this challenge, further lowering the level of detail that can be resolved.

With the continuously increasing quality of cryoEM data and the development of new approaches to data processing, particularly algorithms that allow for non-icosahedral symmetry within a primarily icosahedral structure [52], these challenges can be addressed in the future. Using this approach, one could, for example, visualize longer and heterogeneous ligands that do not adhere to the rules of icosahedral symmetry when binding to virus capsids. Any detailed structural information would drastically increase our understanding of structural mechanisms of virus–GAG binding—an aspect of glycovirology that remains to be more fully explored.
